# Seromuscular Grafts for Bladder Reconstruction: Extra-luminal Demucosalisation of the Bowel

**DOI:** 10.1016/j.urology.2012.07.047

**Published:** 2012-11

**Authors:** Ramnath Subramaniam, Alexander M. Turner, S. Khawar Abbas, David F.M. Thomas, Jennifer Southgate

**Affiliations:** a Department of Paediatric Urology, Leeds Teaching Hospitals NHS Trust, Leeds, United Kingdom; b Jack Birch Unit of Molecular Carcinogenesis, Department of Biology, University of York, York, United Kingdom; c Central Biomedical Services, University of Leeds, Leeds, United Kingdom

## Abstract

**Objective:**

To develop a robust sterile, fully demucosalized and vascularized seromuscular patch for use as an adjunct to novel bioengineering techniques aimed at augmenting, reconstructing, or replacing the bladder because of endstage disease. To eliminate deep colonic epithelial crypts to prevent the possibility of colonocyte regrowth. To maintain sterility by excluding the possibility of contamination from the bowel contents.

**Methods:**

Pilot studies were performed on euthanized pigs to optimize the technique, with tissue samples examined by immunohistochemistry. In vivo, vascularized seromuscular colonic flaps were created from the bowel exterior in 7 large white hybrid pigs. The dissection was facilitated by placing an inflated Foley catheter within the colonic lumen. The seromuscular ends were approximated with 5/0 Vicryl sutures and excess mucosa intussuscepted within the lumen. Demucosalized flaps were used to augment the bladder by composite cystoplasty and were examined immunohistochemically at 3 months.

**Results:**

Pilot studies showed that the technique was successful in creating seromuscular segments with no epithelial remnants. When applied surgically, the seromuscular flaps survived and showed no evidence of colonocyte regrowth at 3 months.

**Conclusion:**

Extraluminal dissection creates robust seromuscular flaps and prevents both regrowth by colonic epithelial cells and contamination of the tissue by exposure to the bowel contents. This technique should find application in a range of bladder reconstruction techniques, including composite cystoplasty and autoaugmentation.

Enterocystoplasty is a well-accepted procedure for bladder augmentation when the bladder is contracted and noncompliant due to a variety of causes. However, it is also associated with long-term consequences of infection and stone formation after mucus production from the interposed enteric mucosa and there is an increasing awareness of the potential for malignancy due to the long-term exposure of the bowel mucosa to urine.^1-4^ Gitlin et al^5^ investigating histologic changes in dogs after enterocystoplasty and gastrocystoplasty noted an overgrowth of a hyperplastic transitional epithelium at the enterovesical and gastrovesical segments, which expressed both uroplakins and mucosubstances. They suggested that the migrated urothelial cells had undergone changes characteristic of enteric and gastric epithelia and that this had implications for the pathogenesis of malignancy in bladder augmentations.

Alternatives to enterocystoplasty have been proposed and include ureterocystoplasty, autoaugmentation, or detrusorectomy with seromuscular colocystoplasty. The latter involves isolating a segment of demucosalized colon and patching it onto the urothelium after detrusorectomy.

The standard technique for demucosalization of the colon is to isolate a segment of the bowel and strip the mucosa from within the lumen.^6-8^ However, one of the significant problems with this technique is colonic epithelial regrowth with its attendant complications of mucus production.^6-8^ Other problems include fibrosis and contraction of the seromuscular flap resulting in failure to achieve an adequate bladder capacity after seromuscular colocystoplasty.^6-8^ Lima et al^9^ described the technique of extraluminal demucosalization and termed it “non secretory colocystoplasty,” reporting good long-term results. In their first report, they isolated a segment of the colon and injected saline between the layers of the mucosa and the submucosa to achieve demucosalization from the exterior.^10^ In subsequent work, they used a Foley catheter inside the lumen to facilitate dissection and then retrieved the mucosa and excised it via the rectum.^11^ Hafez et al^12^ adapted this technique, but instead used hydrodistension of the colonic segment to facilitate the extraluminal dissection. Here, we present results of extraluminal demucosalization with an indwelling Foley catheter. This technique is efficient in preventing colonic regrowth while also preventing contamination of the operative field due to exposure to bowel contents.

## Material and Methods

Previously published human^13^ and porcine^14^ studies have detailed the generation of a functional differentiated urothelium from in vitro-expanded urothelial cells, which displays barrier properties akin to the native bladder. The approach generates adequate urothelium for use in a composite cystoplasty approach, where a demucosalized segment of the colon is combined with autologous urothelial cell sheets grown in the laboratory.^8,15^ This report provides a detailed account of the technique of demucosalization, which is the cornerstone in creating a vascularized flap that precludes colonic mucosal regrowth; application of the technique to composite cystoplasty is reported elsewhere.^15^

Ethical consent was obtained and we adhered to appropriate Home Office procedures. Seven large white hybrid pigs from an original cohort of 11,^15^ which were the subjects of this study, had biopsies performed initially to harvest urothelium for in vitro expansion and differentiation. After recovery (4-6 weeks), the pigs underwent seromuscular colocystoplasty, which was patched with the in vitro-generated autologous urothelium and augmented onto the bladder using a Vicryl mesh as a carrier material (detailed elsewhere^15^). An intravesical balloon (Silimed) was used for 2 weeks to keep the bladder inflated and allow approximation of the urothelium to the flap. The pigs were euthanized 3 months after the augmentation and their bladders were removed for histologic examination, specifically looking for colonic regrowth.

### Technique of Demucosalisation

The abdomen was opened and a self-retaining device placed. A prolene stay suture was placed through the urachus, and the bladder was retracted anteriorly.

**Figure 1:**
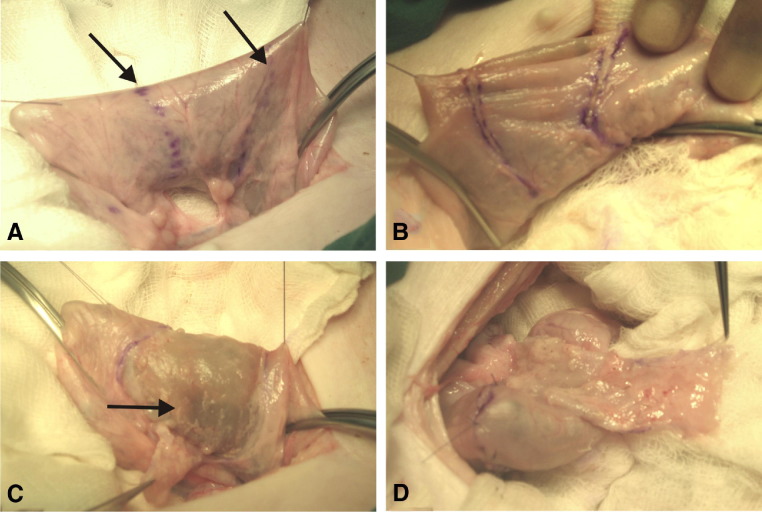
**(A)** Segment of colon identified with marker pen (*arrowed*). **(B)** Extramucosal incisions. **(C)** Seromuscular layer dissected free from underlying mucosa. Note; Foley catheter balloon (*arrowed*) in the lumen helps the dissection by adhering the mucosa to it (mucophilic property). **(D)** De-epithelialized flap with completed anastomosis after intussuscepting the mucosa into the lumen. The mucosa is then shed via the rectum a few days postoperatively.

The sigmoid colon was identified and the segment closest to the posterior bladder wall was surrounded with gauze (Fig. 1). A segment of approximately 10 cm length was identified, along with its vascular arcades within the mesentery. Stay sutures were placed on the antimesenteric seromuscular border of the bowel just beyond the dissection limits. The dissection limits of the bowel and mesentery were defined by marking with a surgical marker pen. Mesenteric fenestrations were made so that there were at least 2 branching vessels supplying the bowel from the vascular arcade. A silicon Foley catheter with a 30 mL balloon was passed per anum and advanced to within the defined sigmoid area. The balloon was filled with sterile water. Using the balloon as a support, the seromuscular layer of the bowel was incised and then, using micro scissors and forceps, the seromuscular layer was separated from the lamina propria deep to the mucosa. At the mesenteric border, the dissection margins were joined with the mesenteric fenestrations to allow the vascularized, de-epithelialized layer to separate from the remaining bowel. The distal leaflet of the pedicled flap was brought through to the bladder side. The inflated balloon acted as a mucophilic device, attracting the mucosa to it and thus facilitating dissection in the plane between the muscularis mucosae and submucosa. The balloon was later deflated and the seromuscular edges from the remaining bowel apposed and closed using 4/0 Vicryl or polydioxanone to “intussuscept” the mucosa.

## Results

**Figure 2:**
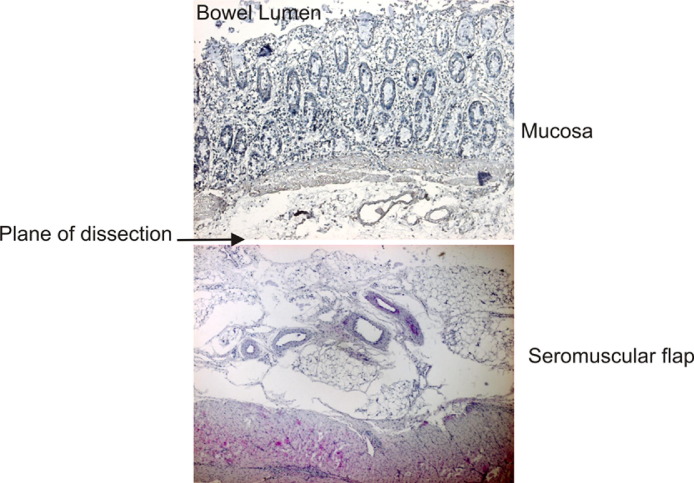
The plane of dissection ran through the submucosa, sparing as many blood vessels as possible. The mucosal specimen showed complete separation from the underlying musculature with no ingress into the crypts. Although it was impossible to recruit all blood vessels to the muscular side, the muscular bed was well vascularized and there was good areolar tissue margin between the muscle and the cut surface.

Pilot studies in euthanized pigs were essential for developing the surgical technique and to demonstrate that the de-epithelialized segment would be suitable for receipt of the tissue-engineered sheet. Histologically, the plane of dissection ran through the submucosa, with the mucosa completely separated from the underlying musculature with no ingress into the crypts. The seromuscular layer was thick and was adequate to receive a urothelial tissue sheet. Although impossible to recruit all blood vessels to the muscular side, the muscular bed was well vascularized and a good areolar tissue margin existed between the muscle and the cut surface (Fig. 2).

The study was completed successfully in the 7 pigs. There was no contamination of the bowel contents intraoperatively and none of the pigs showed any sign of obstruction postoperatively. The latter is particularly relevant as the mucosa was intussuscepted into the lumen, maintaining sterility, and where it was assumed that it would be shed per rectum postoperatively. Notably, we did not have any issues in relation to the excretion per rectum of the sloughed mucosa. Internal and external postmortem examination of the bowel at the site of anastamosis revealed only a scar on the serosal surface.

A continuous epithelium lined all the tissues, both in the native bladder and augmented regions. Morphologically, there was no evidence of colonic crypt regrowth in any of the augmented bladders. The epithelium was transitional in nature in 3 of 7 pigs; in 4 of 7, it appeared of transitional nature, but with some columnar attributes, such as tall, lined-up cells with basally located nuclei. UPK3a expression, unique to the urothelium, was present in all augmented epithelia, confirming a transitional phenotype and that no colonocyte tissue was present.

## Comment

In this study, we have shown that the extraluminal dissection of the colon facilitates the creation of robust seromuscular segments with no colonic epithelial regrowth in a porcine model. The salient feature of our technique is the placement of an intraluminal Foley catheter with the balloon distended to facilitate dissection. This was reported by Lima et al^11^ claiming that this allowed the surgeon to enter the correct plane preventing intestinal regrowth. We entirely agree with their observation and ourselves found that the Foley balloon behaved like a “mucophilic” device attracting the mucosa to it. This allowed us to enter the submucosal plane and prevent leaving islands of mucosal patches that might contribute to intestinal regrowth. Hafez et al^12^ also performed this technique, but with the aid of hydrodistension. They used a catheter to fill the colonic segment with water and then dissect the seromuscular flap. This could potentially cause contamination of the operative field, if there is a mucosal perforation, which can be prevented using a distended Foley balloon.

Another important issue with our technique is that the mucosa was left intact after dissection and, because it was devascularized, it sloughed off and was shed by the natural route. We did not have any case of obstruction in this study. Lima et al^11^ removed the mucosa per rectum and excised it preoperatively.

Dewan et al^6^ observed that enteric mucosal regrowth was a significant problem and suggested removal of the muscularis mucosae with the inner portion of the submucosa to prevent this undesirable complication. However, they stripped the mucosa from within the lumen after isolating the enteric segment. Others have had similar experience with intestinal regrowth in seromuscular colocystoplasty.^7,8^ Badiola et al^16^ used the argon beam coagulator as an adjunct to remove the mucosa and muscularis mucosae from within the lumen. Here we show that the muscularis mucosa remains with the mucosa and not with the submucosa. We believe that extraluminal dissection is a much simpler procedure to prevent this problem.

Lima et al^10^ also used an intravesical balloon, which significantly reduced fibrosis in the canine portion of their study, and commented that in other studies it was the presence of an epithelial lining that seemed to prevent fibrosis and contraction. In our study, we used an intravesical balloon to allow apposition of the epithelial: mesh complex to the seromuscular flap.^15^

We demonstrated that UPK3a was expressed in the epithelium of all our augmented segments, confirming a transitional phenotype with no colonic epithelial regrowth. Although this study from our center, as well as that reported by Hafez et al,^12^ was carried out in pigs,^15^ the seromuscular cystoplasty technique is feasible to do in humans—as already demonstrated by Lima et al^9-11^ and De Badiola et al.^16^

## Conclusions

In conclusion, we suggest that our technique, developed from the lessons of others, is ideal for developing a pure seromuscular template for bladder reconstructive and tissue-engineering applications.
